
JOURNAL INFORMATION
==============================
NLM Title Abbreviation: ScientificWorldJournal
Journal ID: ScientificWorldJournal
NLM Journal ID: 101131163
Journal ID: TSWJ

The Scientific World Journal

ISSN: 2356-6140
EISSN: 1537-744X
Publisher: Wiley

ARTICLE INFORMATION
==============================
PMCID: PMC6084149
PMID: 30147531
DOI: 10.1100/tsw.2001.180
Article ID: 957495
Article version: 1
Subjects: Short Report

Mitochondrial Depolarization During TNF-induced Apoptosis is Caspase-dependent and Independent of Bcl-x L and the Permeability Transition Pore

Cepero E.
Johnson B.W.
Boise L.H.
Publication date: 2001
Electronic publication date: 2001 Nov 30
Volume: 1
First page: 60
Last page: 60
Copyright: Copyright © 2001 E. Cepero et al.
Copyright year: 2001
License: This is an open access article distributed under the Creative Commons Attribution License, which permits unrestricted use, distribution, and reproduction in any medium, provided the original work is properly cited.
License URL: https://creativecommons.org/licenses/by/3.0/

==============================
Miami *Nature Biotechnology* Short Reports
*TheScientificWorld* (2001) 1(S3), 60SR
ISSN 1532-2246; DOI 10.1100/tsw.2001.180

### MITOCHONDRIAL DEPOLARIZATION DURING TNFα-INDUCED APOPTOSIS IS CASPASE-DEPENDENT AND INDEPENDENT OF BCL-$X_L$ AND THE PERMEABILITY TRANSITION PORE

E. Cepero, B.W. Johnson, and L.H. Boise

Dept. of Microbiology and Immunology, University of Miami School of Medicine of Miami School of Medicine, Miami, FL 33101

**INTRODUCTION.** Mitochondria are intimately involved in apoptosis[1] and loss of the mitochondrial transmembrane potential (ΔΨm) has been considered an early event in apoptosis. However, release of cytochrome *c* from the intermembrane space precedes ΔΨm and leads to caspase activation[1]. From this point of view mitochondria can be seen as an initiator of programmed cell death. However, the role of the mitochondrial transmembrane potential and whether it is the cause or the consequence of cell death remains controversial. Our laboratory has shown that TNFα signaling in the IL-3-dependent pro-B cell line FL5.12 can induce apoptosis by sending a type I and a type II signal. In FL5.12 cells caspase 8 can directly activate caspase 3 (type I) and this can be inhibited by zVAD-fmk. The type II signal involves the caspase-8 mediated cleavage of Bid, which is not completely inhibited by zVAD-fmk, proceeds through the mitochondria and is blocked by Bcl-$x_L$. Thus, TNFα-induced apoptosis in FL5.12 cells can be blocked synergistically by Bcl-$x_L$ and zVAD-fmk. The goal of this project is to access the ability of Bcl-$x_L$ to block mitochondrial dysfunction during TNFα-induced apoptosis.

**MATERIALS AND METHODS.** AnnexinV-FITC, propidium iodide staining, and TMRE staining were performed as described[5]. For the cytochrome *c* release assay cells were mechanically lysed in Buffer A and 150 μg of the mitochondrial fraction (10,000 x g pellet) and an equal volume of the cytosol (100,000 x g supernatant) were subjected to western blotting for cytochrome *c*[5]. For DNA isolation, cells lysed in RSB plus 0.5% NP-40, then resuspended in RSB + 2X SDS buffer[5]. Samples were incubated with 400 μg/ml Proteinase K at $50^{o}$C overnight. 1 μg of DNA was run on a 1.5% agarose gel.

**RESULTS.** FL5.12 Neo and Bcl-$x_L$ undergo apoptosis in response to TNFα to the same extent, however differences have been observed at the mitochondrial level. The caspase inhibitor zVAD-fmk has no effect on TNFα-induced cell death in Neo cells but is able to cooperate with Bcl-$x_L$ and block apoptosis. In order to determine whether Bcl-$x_L$ is blocking TNFα-induced mitochondrial dysfunction, determinants of mitochondrial physiology, i.e. ΔΨm and cytochrome *c* release, were examined. After six hours of TNFα-induced apoptosis, in the presence and absence of zVAD-fmk, FL5.12 Neo cells show a loss ΔΨm and release of cytochrome *c*, which is not affected by zVAD-fmk. FL5.12 Bcl-$x_L$ cells, on the other hand, retain their cytochrome *c* within the mitochondria but surprisingly show a loss of ΔΨm. This suggests that cytochrome *c* release and loss of ΔΨm are separate events. Additionally, since TNFα signaling results in loss of ΔΨm, the effects of the permeability transition pore (PTP)

inhibitor Cyclosporin A were assessed. Cyclosporin A alone was unable to prevent loss of $\Delta\Psi m$, suggesting that the PTP is not involved in TNF$\alpha$-induced cell death. Since the combination of zVAD-fmk and Bcl-$x_L$ are required to block $\Delta\Psi m$, the data are most consistent with depolarization occurring at the level of downstream caspases. The mechanism of caspase-activated depolarization is not characterized, however, it is unlikely through opening of the permeability transition pore as Cyclosporin A was unable to prevent mitochondrial depolarization. Taken together, these data suggest that mitochondria can assume two positions in the death pathway. In a mitochondrial-dependent pathway release of cytochrome *c* is required as the initiating event and thus places the mitochondria upstream in the death pathway. In a mitochondrial-independent pathway effector caspases are directly activated and they can target the mitochondria for inactivation, just as they target the genome and the cytoskeleton, thus placing the mitochondria downstream in the death pathway.

**REFERENCES.**

1. Green, D.R. and Reed, J.C. (1998) Science 281,1309-1312
2. Bossy-Wetzel, E., Newmeyer, D.D., and Green, D.R. (1998) EMBO J. 17, 37-49
3. Johnson, B.W. and Boise, L.H. (1999) J. Biol. Chem. 274, 18552-18558
4. Johnson, B.W., Cepero, E., and Boise, L.H. (2000) J. Biol. Chem. 275, 31546-31553
